# Extracts of *Euphorbia hirta* Linn. (Euphorbiaceae) and *Rauvolfia vomitoria* Afzel (Apocynaceae) demonstrate activities against *Onchocerca volvulus* Microfilariae *in vitro*

**DOI:** 10.1186/1472-6882-13-66

**Published:** 2013-03-18

**Authors:** Simon K Attah, Patrick F Ayeh-Kumi, Archibald A Sittie, Isaac V Oppong, Alexander K Nyarko

**Affiliations:** 1 Department of Microbiology, University of Ghana Medical School, University of Ghana, Legon, Ghana; 2 Centre for Scientific Research into Plant Medicine, Mampong-Akwapem, Ghana; 3 Chemistry Department, University of Ghana, Legon, Accra, Ghana; 4 Noguchi Memorial Institute for Medical Research, University of Ghana, Legon, Ghana

**Keywords:** *Euphorbia hirta*, *Rauvolfia vomitoria*, *Onchocerca volvulus*, Microfilariae, *In vitro*

## Abstract

**Background:**

Onchocerciasis transmitted by *Onchocerca volvulus* is the second major cause of blindness in the world and it impacts negatively on the socio-economic development of the communities affected. Currently, ivermectin, a microfilaricidal drug is the only drug recommended for treating this disease. There have been speculations, of late, concerning *O. volvulus* resistance to ivermectin. Owing to this, it has become imperative to search for new drugs. World-wide, ethnomedicines including extracts of *Euphorbia hirta* and *Rauvolfia vomitoria* are used for treating various diseases, both infectious and non-infectious.

**Method:**

In this study extracts of the two plants were evaluated *in vitro* in order to determine their effect against *O. volvulus* microfilariae. The toxicity of the *E. hirta* extracts on monkey kidney cell (LLCMK2) lines was also determined.

**Results:**

The investigations showed that extracts of both plants immobilised microfilariae at different levels *in vitro* and, therefore, possess antifilarial properties. It was found that all the *E. hirta* extracts with the exception of the hexane extracts were more effective than those of *R. vomitoria*. Among the extracts of *E. hirta* the ethyl acetate fraction was most effective, and comparable to that of dimethanesulphonate salt but higher than that of Melarsoprol (Mel B). However, the crude ethanolic extract of *E. hirta* was found to be the least toxic to the LLCMK2 compared to the fractionated forms.

**Conclusions:**

Extracts from both plants possess antifilarial properties; however, the crude extract of *E. hirta* was found to be least toxic to LLCMK2.

## Background

Onchocerciasis, transmitted by *Onchocerca volvulus* is among the major causes of blindness in the world [1-4] and has a negative impact on the socio-economic development of the communities affected [5-12]. The pathology of the disease involves mainly the microfilariae (mff) of the parasite. Ivermectin (Mectizan®), the drug recommended for treatment is microfilaricidal only and has to be administered once within six to 12 months and continuously for several years [13-18]. Of late, there have been reports of emerging ivermectin resistance in the adult *O. volvulus*[19-24]. Should this be confirmed, the control of the disease will seriously be in jeopardy. In view of the limited candidate drugs available for testing against the parasite, it has become imperative to search for new drug(s) preferably a macrofilaricide(s) to treat the disease.

Presently, one area that is gaining grounds and acceptability world-wide is the evaluation and development of compounds from medicinal plants for the treatment of diseases. World-wide, ethnomedicines are used for treating various diseases, both infectious and non-infectious. In Ghana, a number of plant based products are claimed to be effective for the treatment of onchocerciasis. Among the medicinal plants involved *Euphorbia hirta* and *Rauvolfia vomitoria* reported to have antimicrobial properties [25-32] are used for this purpose. These have however, not been subjected to systematic evaluation to ascertain their effectiveness against *O. volvulus*. Owing to this, extracts from these two plants were selected for *in vitro* evaluation against the microfilariae (mff) of *O. volvulus.* The toxicity of the *E. hirta* extracts on monkey kidney cell (LLCMK2) lines was also determined following satisfactory results by testing its extracts.

## Methods

### Plant collection, identification and extract preparation

Whole plants of *E. hirta* and the leaves and roots of *R. vomitoria* were collected and identified at the herbarium of the Botany Department of the University of Ghana in Accra, Ghana. Voucher specimens of the plants: *E. hirta* (GC47751) and *R. vomitoria* (GC47752) were deposited at the herbarium. Whole plant specimens of *E. hirta* and root specimens of *R. vomitoria* were air dried for a week and pulverized into a fine powder. About 300 g of the pulverized *E. hirta* and 270 g of the *R. vomitoria* materials were macerated separately in three litres of 80% ethanol. After 24 hours the slurry of each plant was filtered with Whatman No.1 filter paper and concentrated under reduced pressure in a rotavapor (BUCHI Rotavapor R-114, Switzerland) at 50°C to recover the ethanol.

A portion of the concentrate was kept as crude extract. The remaining concentrate was partitioned with hexane (3 × 250 ml), chloroform (3 × 250 ml) and ethyl acetate (3 × 250 ml) sequentially (i.e. in an increasing order of polarity). Each fraction was then concentrated *in vacuo* at 50°C with the rotavapor to yield semi-solid masses. The residual (aqueous) solutions as well as the crude extracts were freeze-dried. All the extracts were stored at −20°C until they were used for testing.

Dimethylsulfoxide (DMSO) was used to dissolve the plant extracts prior to diluting them into stock concentrations of 1000 μg/ml in MEM. Prior to testing against the mff, concentrations ranging from 6.25 to 800 μg/ml of each of the extracts were prepared from the working concentration such that the concentration of DMSO in each of the test solution was less than 0.1%. Melarsoprol (Mel B) and dimethanesulphonate (DMSPN) salt (Ash Stevens Inc. Detriot, Michigan 48202) used as reference drugs (Strote *et al*., 1990a and b, 1997 and 1998) were prepared similarly.

For testing against monkey kidney cell lines (LLCMK2) 1:10 serial dilutions of the *E. hirta* stock solutions were prepared down to 1 μg/ml. Potassium cyanide (KCN) (at 1 mg/ml of MEM) solution was used as a positive control and 1% DMSO in MEM as a solvent (or negative) control. All the solutions were sterile filtered with a 0.2 μm millipore filter before testing.

### Selection of Subjects

Twenty-four onchocerciasis subjects between the ages of 18 and 60 from Kpedze-Anoe and Honuta-Gbogame, who had not taken any antifilarial drugs before were admitted to the Onchocerciasis Chemotherapy Research Centre (OCRC) at Hohoe in Ghana after a written consent had been sought from each of them. The study was carried out between May 5 and August 25, 2005 following approval from the Ethical Review Board of the Noguchi Memorial Institute for Medical Research, University of Ghana.

### Harvesting of microfilariae

Skin snips were taken from both iliac crests after sterilizing these sites with 70% alcohol. The snips were placed in a sterile petri-dish containing five millilitres of Eagle’s Minimum Essential Medium (MEM) and left on the bench at 22°C. After three hours of incubation, the snips were removed, medium transferred into sterile test tubes and the mff recovered by centrifugation at 1,500 rpm.

### Measurement of microfilaricidal action of extracts

One millilitre of each extract and drug preparations was delivered in triplicates into a 24-well plastic plate. About 30 mff were delivered into each well. The number of motile mff was determined at 2, 4, 6, 12, 24 and 48 hours of exposure to the extracts. The same amount of mff was exposed to concentrations between 0.01 and 1.0 per cent of DMSO (solvent control) and in plain MEM (i.e. without DMSO, drug or herbal preparation). The assays were carried out in triplicates. Microfilariae were observed under an inverted microscope and the proportion of motile/live ones determined.

### Inhibition determination of *Euphorbia hirta* extracts on monkey kidney cells

Three hundred microlitres of MEM solution containing about 1000 monkey kidney cells were placed in each well of 96-well plates. The cells were incubated at 35°C in a humidified atmosphere of 5% CO_2_ for a day. At the beginning of day two the culture medium was removed from the wells and replaced with 300 μl of the different diluted extract to give a concentration of 0–1000 μg/ml and left to incubate for five days. The same volume of potassium cyanide (KCN) solution at 1 mg/ml concentration and DMSO in MEM were each delivered into three wells of the plate. The cells in three of the wells were killed with hot water and incubated with plain MEM culture medium (i.e., without any extract). In three other wells the cells were maintained with the plain culture medium. In other experiments, the cells were incubated for four days before the plant extracts were added and incubated for another two days.

At the end of the incubation period the plant extracts were removed from the wells and replaced with 300 μl of tetrazolium salt, 3-(4, 5 dimethylthiazol-2-yl)-2, 5-diphenyltetrazolium bromide (MTT) solution at 0.5 mg/ml and incubated at 35°C. After 30 minutes the MTT solution was removed and replaced with 100 μl DMSO and the plate placed in the dark at a temperature of 22°C for 1 hour. The formazan produced in each of the wells was transferred into corresponding wells of another microtitre plate in duplicates and the optical density (OD) determined spectrophotometrically at a wavelength of 510 nm.

### Data management and statistical analysis

Data were analyzed using Excel 2000 and Epi-info 6 programme version 6.04d, January 2001. The 50% inhibition concentrations (IC_50s_) of the extracts were determined by log probit analysis, and the percentage inhibitions of the extracts on LLCMK2 were calculated using the means of the OD values. For comparison of results the chi-square test was used.

## Results

### Effect of DMSO on microfilariae

All the mff maintained in the various concentrations of the solvent control retained their viability up to six hours of exposure. At 12 hours of exposure, there was a slight decrease in the number of live/motile mff in the 0.5% concentration (i.e. a decrease of 6.4%) and 1% concentration (i.e. a decrease of 1.8%). The mff maintained their survival above 90% at 24 hours and between 69% and 76% at 48 hours in all the concentrations. At 48 hours of exposure about 33% of the mff were immobilised in the 1% DMSO concentration and about 22% in the plain MEM. No significant difference was found between the proportions of mff immobilised in these two solutions.

### Effect of the plant extracts on microfilariae

The results from the investigations showed that motile mff were immobilised in various degrees by the *E. hirta* and *R. vomitoria* extracts in a concentration and time dependent fashion. At 2 hrs of mff exposure more mff were already immobilised in the 400–800 μg/ml of the crude extract, 800 μg/ml of the hexane, 400 μg/ml and 800 μg/ml of the chloroform, 25–800 μg/ml of the ethyl acetate and 400 μg/ml and 800 μg/ml of the aqueous extracts of the *E. hirta* plant than the 1% DMSO concentration (p < 0.001). Immobilisation of mff continued in all the concentrations of the *E. hirta* extracts (with the exception of the 6.25 μg/ml and 12.5 μg/ml concentrations of the hexane extracts) and the reference drugs so much so that by 48 hrs, a significant difference was found between the proportions of mff that were immobilised in those concentrations and in the 1% DMSO concentration (p < 0.001). For the *R. vomitoria* extracts more mff were found immobilised in the 400 μg/ml and the 800 μg/ml concentrations than in the 1% DMSO (p < 0.001) from 2 hrs to 24 hrs. Similarly, with the exception of the 6.25 μg/ml of the crude extract and the 6.25-50 μg/ml of the hexane and ethyl acetate extracts, more mff were found immobilised in the lower concentrations (6.25-200 μg/ml) of the extracts of this plant than in the 1% DMSO concentration at 48 hrs (p < 0.001).

Table 1 shows the percentages of motile *O. volvulus* mff found in different concentrations of extracts of the two plants ranging between 6.25 μg/ml and 200 μg/ml at 2 and 4 hrs. The minimum concentrations of *E. hirta* found to have immobilised the mff by 2 hrs of exposure were: the 12.5 μg/ml concentration of the ethyl acetate, 100 μg/ml of the aqueous, 200 μg/ml of the crude, 400 μg/ml of the chloroform and 800 μg/ml of the hexane extracts*.* The least concentration that reduced motile mff numbers to less than 50% among the *E. hirta* extracts was the 200 μg/ml concentration of the ethyl acetate extract. Reduction of live mff below 50% also occurred in the 400 μg/ml of the aqueous extract and in the crude and chloroform extracts at 800 μg/ml concentrations of that plant (Table 2). The various concentrations of the hexane fraction were the least active among the *E. hirta* extracts. No significant reduction of mff was seen in all the concentrations of the *R. vomitoria* extracts except in the 800 μg/ml chloroform and the ethyl acetate extracts (Table 2).

**Table 1:** **Percentage (± SE) of motile *Onchocerca volvulus *microfilariae found in 6.25 μg/ml to 200 μg/ml of *Euphorbia hirta *and *Rauvolfia vomitoria *extracts at 2 and 4 hrs**

**Extract/drug**	**Extract concentration(μg/ml)**											
	**6.25 (μg/ml)**		**12.5 (μg/ml)**		**25 (μg/ml)**		**50 (μg/ml)**		**100 (μg/ml)**		**200 (μg/ml)**	
	**2 hrs**	**4 hrs**	**2 hrs**	**4 hrs**	**2 hrs**	**4 hrs**	**2 hrs**	**4 hrs**	**2 hrs**	**4 hrs**	**2 hrs**	**4 hrs**
***Euphorbia hirta***												
Crude	100	100	100	97(±3)	100	95(±5)	100	90(±10)	100	55(±29)	73(±24)	36(±32)
Hexane	100	100	100	100	100	100	100	100	100	100	100	100
Chloroform	100	100	100	97(±3)	100	92(±8)	100	69(±22)	100	71(±22)	100	32(±21)
Ethyl acetate	100	98(±2)	98(±2)	96(±2)	64(±28)	54(±27)	55(±29)	46(±23)	54(±29)	38(±28)	35(±19)	2(±2)
Aqueous	100	100	100	100	100	100	100	100	96(±4)	62(±25)	94(±4)	43(±28)
***Rauvolfia vomitoria***												
Crude	100	100	100	100	100	100	100	100	100	100	100	90(±10)
Hexane	100	100	100	100	100	100	100	100	100	100	100	100
Chloroform	100	100	100	100	100	100	100	100	100	100	100	98(±2)
Ethyl acetate	100	100	100	100	100	100	100	100	100	100	100	100
Aqueous	100	100	100	100	100	100	100	100	100	100	100	100
**Reference drugs**												
DMSPN	100	100	100	92(±8)	97(±3)	70(±20)	67(±33)	38(±27)	49(±28)	17(±12)	44(±28)	14(±8)
Mel B	100	100	100	100	100	99(±1)	100	99(±1)	100	99(±1)	94(±6)	81(±11)

**Table 2:** **Percentage (± SE) of motile *Onchocerca volvulus *microfilariae found in 400 μg/ml and 800 μg/ml of *Euphorbia hirta *and *Rauvolfia vomitoria *extracts at 2 to 48 hrs**

**Extract/drug**	**Extract concentration(μg/ml)**											
	**400**	**800**	**400**	**800**	**400**	**800**	**400**	**800**	**400**	**800**	**400**	**800**
	**2 hrs**		**4 hrs**		**6 hrs**		**12 hrs**		**24 hrs**		**48 hrs**	
***Euphorbia hirta***												
Crude	55(±24)	27(±23)	31(±26)	12(±12)	2(±2)	0	0	0	0	0	0	0
Hexane	100	64(±32)	87(±13)	33(±33)	87(±13)	0	43(±20)	0	4(±4)	0	0	0
Chloroform	50(±17)	12(±9)	17(±17)	10(±1)	0	0	0	0	0	0	0	0
Ethyl acetate	24(±24)	10(±10)	2(±2)	2(±2)	0	0	0	0	0	0	0	0
Aqueous	46(±27)	32(±21)	28(±18)	24(±24)	2(±2)	0	0	0	0	0	0	0
***Rauvolfia vomitoria***												
Crude	88(±12)	88(±12)	48(±22)	33(±27)	42(±27)	33(±33)	30(±30)	0	30(±30)	0	22 (±22)	0
Hexane	98(±2)	87(±13)	96(±2)	33(±33)	96(±2)	0	70(±18)	0	21(±5)	0	8 (±8)	0
Chloroform	80(±15)	30(±10)	76(±13)	17(±17)	59(±30)	6(±6)	56(±28)	0	53(±27)	0	0	0
Ethyl acetate	100	12(±12)	97(±3)	0	97(±3)	0	94(±3)	0	60(±30)	0	7(±7)	0
Aqueous	100	100	88(±9)	67(±33)	88(±9)	42(±30)	65(±32)	23(±23)	59(±30)	23(±23)	30 (±30)	0
**Reference drugs**												
DMSPN	44(±28)	15(±15)	11(±9)	0	0	0	0	0	0	0	0	0
Mel B	88(±5)	3(±3)	65(±11)	0	55(±2)	0	0	0	0	0	0	0

NB: in 1% DMSO 99%, 93.1% and 68% of mff were motile at 12 hrs, 24 and 48 hrs of exposure respectively.

Among the 6.25 μg/ml concentrations of *E. hirta* immobilisation of mff occurred only in the ethyl acetate extract at 4 hours, and also occurred in the chloroform extract at 6 hrs and aqueous extracts at 12 hrs. At 4 hrs the least concentration of each of the *E. hirta* extract that reduced motile mff numbers to less than 50% was 50 μg/ml of the ethyl acetate extract, 200 μg/ml of the crude extract, the chloroform extract and the aqueous extract and 800 μg/ml of the hexane extract. At this time point, the proportion of motile mff had been reduced significantly to 2% in the 200 μg/ml concentration of the *E. hirta* ethyl acetate extract. There was no significant reduction of motile mff numbers in the concentrations of *R. vomitoria* extracts up to 200 μg/ml at this time point (Table 1).

Table 3 shows the percentage of motile *O. volvulus* mff found in concentrations of extracts of the two plants ranging between 6.25 μg/ml and 200 μg/ml at 6 and 12 hrs. At 6 hours of exposure to concentrations of the hexane extracts of *E. hirta* ranging between 6.25 μg/ml and 200 μg/ml no mff was found immobilised. Immobilisation of mff was observed in the 12.5 μg/ml concentrations of the remaining extracts of this plant. The reduction of motile mff numbers occurred gradually in the crude, chloroform and aqueous extracts of *E. hirta* but the rate of reduction was faster in the ethyl acetate extract of this plant. The minimum concentration that immobilised more than 90% of the mff at this time point was the 100 μg/ml of the ethyl acetate, 400 μg/ml of the chloroform, aqueous and crude extracts and 800 μg/ml of the hexane extract (Table 2). No significant difference was found between the effect of the aqueous extract and that of the chloroform or the crude extracts. A significant difference was however found between the effectiveness of the aqueous extract and that of the hexane extract (P < 0.001). In the case of the *R. vomitoria* extracts, no significant reduction of live mff numbers was observed except in the 400 μg/ml concentration of the crude extract and the 800 μg/ml concentration of the other extracts up to this time point of mff exposure (Table 2).

**Table 3:** **Percentage (± SE) of motile *Onchocerca volvulus *microfilariae found in 6.25 μg/ml to 200 μg/ml of *Euphorbia hirta *and *Rauvolfia vomitoria *extracts at 6 and 12 hrs**

**Extract/drug**	**Extract concentration(μg/ml)**											
	**6.25 (μg/ml)**		**12.5 (μg/ml)**		**25 (μg/ml)**		**50 (μg/ml)**		**100 (μg/ml)**		**200 (μg/ml)**	
	**6 hrs**	**12 hrs**	**6 hrs**	**12 hrs**	**6 hrs**	**12 hrs**	**6 hrs**	**12 hrs**	**6 hrs**	**12 hrs**	**6 hrs**	**12 hrs**
***Euphorbia hirta***												
Crude	100	100	95(±5)	93(±4)	95(±5)	93(±4)	83(±9)	50(±25)	15(±13)	4(±4)	13(±10)	4(±4)
Hexane	100	100	100	100	100	100	100	100	100	85(±1)	100	63(±18)
Chloroform	100	94(±6)	94(±6)	94(±6)	92(±8)	82(±11)	67(±26)	42(±23)	67(±26)	14(±14)	22(±12)	10(±5)
Ethyl acetate	96(±4)	77(±6)	85(±8)	77(±6)	39(±24)	10(±6)	20(±10)	0	2(±2)	0	2(±2)	0
Aqueous	100	83(±10)	97(±3)	82(±10)	85(±11)	50(±26)	73(±18)	50(±26)	47(±27)	18(±10)	29(±29)	8(±8)
***Rauvolfia vomitoria***												
Crude	100	100	100	100	100	100	100	93(±7)	96(±4)	83(±17)	76(±24)	67(±33)
Hexane	100	100	100	100	100	100	100	100	100	100	100	95(±5)
Chloroform	100	100	100	98(±2)	100	98(±2)	100	97(±3)	100	97(±3)	98(±2)	65(±33)
Ethyl acetate	100	100	100	100	100	100	100	100	100	100	100	100
Aqueous	100	96(±4)	100	100	100	100	100	100	100	100	100	91(±6)
**Reference drugs**												
DMSPN	100	84(±16)	64(±18)	55(±23)	38(±26)	19(±16)	33(±24)	10(±10)	11(±9)	10(±10)	5(±5)	5(±5)
Mel B	94(±6)	87(±8)	94(±6)	87(±10)	94(±6)	87(±8)	94(±6)	64(±14)	94(±6)	43(±23)	71(±21)	10(±5)

In the 6.25 μg/ml concentration of the *E. hirta* extracts all the mff were motile in the crude and hexane extracts up to 12 hours of their exposure. At this time point, no live mff were found in concentrations higher than 25 μg/ml of the ethyl acetate extract. Live mff numbers fell below 20% in the 100 μg/ml and 200 μg/ml concentrations, and fell to zero in the 400 μg/ml and 800 μg/ml concentrations of the crude, chloroform and aqueous extracts. In the *R. vomitoria* extracts all the mff were motile up to 12 hours when they were exposed to the 6.25 μg/ml concentration. No mff was found immobilised in the hexane extracts of *E. hirta* at concentrations lower than 400 μg/ml except in the 100 μg/ml and 200 μg/ml concentrations.

Table 4 shows the percentage of motile mff exposed to the 6.25 μg/ml to 200 μg/ml concentrations of *E. hirta* and *R. vomitoria* extracts at 24 hrs and 48 hrs and Table 2 shows the percentage of motile mff exposed to the 400 μg/ml and 800 μg/ml concentrations of *E. hirta* and *R. vomitoria* extracts at 2–24 hrs. At 24 hrs of exposure to the *E. hirta* extracts the proportion of motile mff were found to have been reduced drastically. The lowest concentration that killed all the mff at this time point in the case of the ethyl acetate extract was 25 μg/ml; chloroform extract, 100 μg/ml (Table 4) and aqueous extract, 400 μg/ml; hexane extract, 800 μg/ml and the crude extract, 400 μg/ml (Table 2). In all the plant extract concentrations, only in the 800 μg/ml concentration of the aqueous extract of *R. vomitoria* were motile mff seen at the same time point (Table 2). By 48 hrs, all the mff were immobilised in all the concentrations of the ethyl acetate extract of *E. hirta* and in the 800 μg/ml concentrations of both the *E. hirta* and *R. vomitoria* extracts (Tables 2 and 4). At this time point, with the *E. hirta* plant, motile mff were found in the 6.25 μg/ml and 12.5 μg/ml concentrations of the aqueous extract and the 6.25 μg/ml to 25 μg/ml concentrations of the chloroform and crude extracts and 6.25-200 μg/ml of the hexane extract.

**Table 4:** **Percentage (± SE) of motile *Onchocerca volvulus *microfilariae found in 6.25 μg/ml to 200 μg/ml of *Euphorbia hirta *and *Rauvolfia vomitoria *extracts at 24 and 48 hrs**

**Extract/drug**	**Extract concentration(μg/ml)**											
	**6.25 (μg/ml)**		**12.5 (μg/ml)**		**25 (μg/ml)**		**50 (μg/ml)**		**100 (μg/ml)**		**200 (μg/ml)**	
	**24 hrs**	**48 hrs**	**24 hrs**	**48 hrs**	**24 ± hrs**	**48 hrs**	**24 hrs**	**48 hrs**	**24 hrs**	**48 hrs**	**24 hrs**	**48 hrs**
***Euphorbia hirta***												
Crude	73(±19)	34(±25)	67 (±21)	34(±25)	67(±21)	25(±25)	35 (±22)	0	4(±4)	0	4(±4)	0
Hexane	96(±4)	62(±20)	96(±4)	54(±23)	78(±13)	44(±1)	74(±1)	44(±1)	74(±1)	33(±1)	38(±31)	12(±12)
Chloroform	77(±12)	33(±33)	63(±23)	33(±33)	63(±22)	25(±25)	20(±20)	0	0	0	0	0
Ethyl acetate	27(±27)	0	27(±27)	0	0	0	0	0	0	0	0	0
Aqueous	65(±19)	33(±33)	62(±19)	33(±33)	39(±19)	0	23(±17)	0	10(±5)	0	8(±8)	0
***Rauvolfia vomitoria***												
Crude	92(±1)	33(±33)	92(±1)	33(±33)	92(±1)	33(±33)	87(±13)	33(±33)	71(±29)	33(±33)	67(±33)	33(±33)
Hexane	89(±11)	33(±33)	89(±11)	33(±33)	80(±1)	33(±33)	72(±20)	33(±33)	71(±20)	33(±33)	46(±27)	33(±33)
Chloroform	100	33(±33)	97(±3)	33(±33)	97(±3)	33(±33)	97(±3)	33(±33)	97(±3)	33(±33)	64(±22)	0
Ethyl acetate	100	67(±33)	100	67(±33)	100	63(±32)	100	33(±33)	67(±33)	33(±33)	67(±33)	33(±33)
Aqueous	100	33(±33)	100	33(±33)	100	33(±33)	79(±18)	33(±33)	78(±22)	33(±33)	59(±30)	30(±30)
**Reference drugs**												
DMSPN	49(±26)	17(±17)	25(±25)	8(±8)	3(±3)	0	3(±3)	0	3(±3)	0	2(±2)	0
Mel B	22(±22)	17(±17)	22(22)	0	6(±6)	0	6(±6)	0	0	0	0	0

Generally, the extracts of *E. hirta* were more effective in immobilising the mff and their IC_50s_ were lower than those of *R. vomitoria*. At 2–24 hrs the extracts of *E. hirta* were found to be more effective in immobilising mff than those of *R. vomitoria* and the difference was significant (P < 0.004). There was no significant difference between the ethyl acetate extract of *E. hirta* and DMSPN at any time point with regards to their effectiveness. The various concentrations of Mel B were less effective in immobilising motile mff than those of the ethyl acetate extract of *E. hirta* (P < 0.019) and those of DMSPN (P < 0.01) at 2–6 hrs (Tables 1 and 3). Table 5 shows the 50% inhibition concentrations (IC_50s_) of the plant extracts and the reference drugs on *O. volvulus* mff. The IC_50s_ of all the extracts and the reference drugs decreased consistently over time. The *E. hirta* extracts with the exception of that of the hexane had lower IC_50s_ than those of *R. vomitoria* at 24 hrs and Mel B at 12 hrs. Among the *E. hirta* extracts that of ethyl acetate had the least IC_50s_ which were comparable to those of DMSPN up to 24 hrs. With the exception of the hexane extract of *E. hirta* and the ethyl acetate extract of *R. vomitoria*, the IC_50s_ of all the extracts as well as DMSPN and Mel B fell below 6.25 μg/ml at 48 hrs. From the foregoing, it can be deduced that the *E. hirta* ethyl acetate extract (which had the least IC_50s_) was the most effective.

**Table 5:** **Fifty per cent inhibition concentrations (IC**_**50s**_**) of *Euphorbia hirta *and *Rauvolfia vomitoria *extracts on *Onchocerca volvulus *microfilariae**

**Extract/Drug**	**Time of mff exposure (hrs)**					
	**2**	**4**	**6**	**12**	**24**	**48**
**IC**_**50s **_**of *Euphorbia hirta *(μg/ml)**						
**Crude**	500	125	75	50	40	<6.25
**Hexane**	>800	700	700	700	150	17.5
**Chloroform**	400	150	140	50	20	<6.25
**Ethyl acetate**	100	37.5	21.9	12.5	<6.25	<6.25
**Water**	400	150	90	25	18.8	<6.25
**IC**_**50s **_**of *Rauvolfia vomitoria *(μg/ml)**						
**Crude**	>800	400	350	300	300	<6.25
**Hexane**	>800	700	600	450	200	<6.25
**Chloroform**	650	600	450	400	400	<6.25
**Ethyl acetate**	650	625	625	625	450	37.5
**Water**	>800	>800	800	400	200	<6.25
**IC**_**50s **_**of the reference drugs (μg/ml)**						
**DMSPN**	100	40.5	18.8	14.1	6.25	<6.25
**Mel B**	600	500	400	87.5	<6.25	<6.25

### Effect of *E. hirta* extracts on monkey kidney cell lines

**Figure 1:**
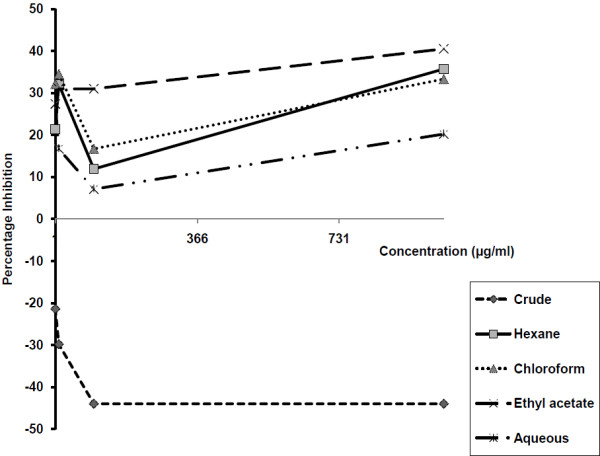
**Percentage inhibition of monkey kidney cells lines maintained in culture for one day before exposure to *Euphorbia hirta *extracts for 5 days**^**1**^**.** 1. Percentage inhibition of potassium cyanide (KCN) at 10^3^ μg/ml = 131%; the optical density (OD) values for: 1% DMSO (solvent control) = 0.069, cells cultured in plain culture medium = 0.093, and for heat killed cells = −0.015 (negative figures imply promotion of cell maintenance).

**Figure 2:**
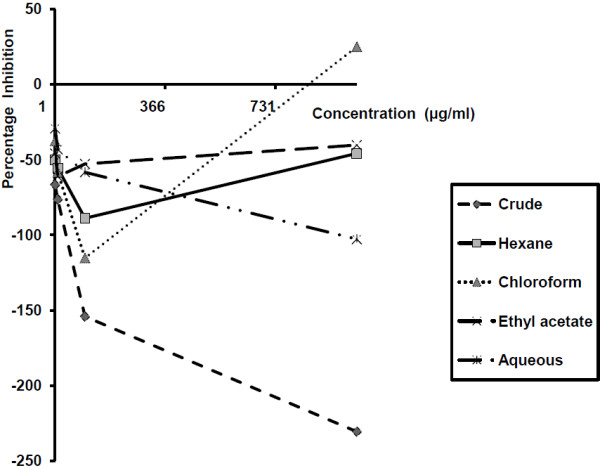
**Percentage inhibition of monkey kidney cells lines maintained in culture for four days before exposure to *Euphorbia hirta *extracts for two days**^**2**^**.** 2. Percentage inhibition of potassium cyanide (KCN) at 10^3^ μg/ml = 52.8%; the optical density (OD) values for 1% DMSO (solvent control) = 0.109, cells cultured in plain culture medium = 0.149, and for heat killed cells = −0.037 (negative figures imply promotion of cell maintenance).

Figure 1 shows the effect of *E. hirta* extracts on the viability of monkey kidney cells maintained for one day prior to the exposure of the extracts for five days. In this figure the crude extracts were seen to exhibit negative inhibition on the LLCMK2. All the extracts with the exception of that of chloroform at 1000 μg/ml concentration also inhibited the cells negatively (i.e. promoted cell proliferation) when they were maintained in culture for four days before being exposed to the extracts for two days (Figure 2).

Among these extracts only the crude extract inhibited the cells in a concentration dependent fashion with the lowest concentration having the highest effect and vice versa. At each concentration the crude extract exhibited the lowest inhibition on the cells. The 1000 μg/ml concentration of the hexane extract showed the highest inhibition and the 100 μg/ml the lowest in these two experiments. In the first experiment the highest inhibition on the cells was exhibited by the 10 μg/ml, the 1000 μg/ml and the 1 μg/ml concentrations of the chloroform, ethyl acetate and water respectively whilst the 100 μg/ml, 1 μg/ml, and 100 μg/ml concentrations in the same order had the lowest effect on the cells (Figure 1). In the second experiment the 1000 μg/ml concentration of the chloroform and ethyl acetate extract as well as the 1 μg/ml concentration of the aqueous extract had the highest whilst the 100 μg/ml concentrations of the chloroform, ethyl acetate and aqueous extracts respectively had the lowest inhibitory effect on the cells (Figure 2).

## Discussion

To date, not much work has been done to investigate the efficacy of plant extracts against human filarial parasites except those conducted by Kilian *et al*. (1990) using *Cassia aubrevillei*[33] and Titanji *et al*. (1990) using *Polyalthia suaveolens*[34]. Most of the work conducted so far involved the use of animal filarial parasites. Some of the plants used were *Acacia auriculiformis*[35], *Sancio nudicaulis* Buch. Ham. [36], *Saxifraga stracheyion* root [37], *Centratherum anthelminticum* seeds [38], *Azadirachta indica* and *Ficus racemosa* Linn. [39,40]. Comley *et al*. (1990) reported the macrofilaricidal effects *in vitro* of filaricid (a crude extract of the stem bark of *Streblus asper*, Lour) and filarin (a crude extract comprising five plants in different proportions) against *Acanthocheilonema viteae* females at 500 μg/ml [41]. They also found that oliverine derived from n-hexane extract of the stem bark of *Pachypodanthium staudtii* has similar effects on the *Acanthocheilonema viteae* worm at 120 hours down to 10 μg/ml.

In spite of reports concerning *E. hirta* and *R. vomitoria* possessing antimicrobial properties, no systematic studies on their effectiveness against filarial parasites either *in vivo* or *in vitro* have been carried out. The results of this study has indicated that extracts of these two plants possess antifilarial properties. Among the *E. hirta* extracts, that of ethyl acetate was most effective, and this was comparable to that of DMSPN but more effective than Mel B. The hexane extract of *E. hirta* and the extracts of *R. vomitoria* showed least activity before 48 hours of mff exposure. A lot of chemical constituents including diterpenes, flavonoids, tannins, aromatic acids, alkaloids, coumarins and anthocyanins, euphorbon, triterpenes and quercetol have been isolated from every part of *E. hirta*[28,29]. The root of *R. vomitoria*, on the other hand, contains flavonoids and over 100 alkaloids including reserpine, rescinnamine, deserpidine, yohimbine, aijmaline and alstonine which are therapeutically important [27-29].

Notwithstanding the efficacy of *E. hirta* extracts against these diseases, issues have been raised concerning the toxicity of the extracts. For instance, Adedapo *et al*. (2005) in conducting *in vivo* work showed that some chromatographic fractions of the plant have potentially deleterious effect on the serum chemistry of rats. They therefore cautioned against the use of this plant for medicinal purposes. Contrary to these findings, Hashemi *et al.* (2008) observed a significant decrease in Aspartate transaminase (AST), Alanine aminotransferase (ALT), and Alkaline phosphatase (ALP) after 14 days of oral administration of 2000 mg/kg of the crude aqueous extracts of *E. hirta* to chicks [42]. Ogueke *et al.* (2007) found the crude ethanolic extracts of the plant at concentrations ranging from 60.4 to 483.0 mg/kg body weight to be haematologically safe to albino rats. Biochemical analyses of the renal and hepato-biliary function following oral administration of up to 3000 mg/kg body weight of the crude extracts of the plant were also found to be haematologically safe and did not show any signs of nephrotoxicity and hepatotoxicity in rats, as confirmed by histopathological examination [43].

*E. hirta* has been used to treat various diseases by traditional herbal practitioners and its administration has mainly been through the oral route [25,28-30]. In this study, the crude extract of *E. hirta* inhibited cell growth the least. Moreover, it was the only extract that was found to have exhibited its effect in a concentration dependent fashion with the highest concentration having the least inhibitory effect. This observation is in agreement with that of Brindha *et al.* (2010) who observed a dose-dependent increase in viability when antitubercular drug exposed human liver derived HepG2 cells were treated with different concentrations of extracts of the plant [44].

In the current study, when the cells were maintained for a longer period before being exposed to the extracts, the survival of the cells was enhanced more than when they were maintained for a shorter period before their exposure to the extracts. This observation was most pronounced when the cells were exposed to the crude extracts. This observation can be explained by the fact that once the cells form confluence they are inhibited less than when they have not. A previous investigation by Raja Sidambaram *et al*. (2011) using the methanol extract of the leaves of *E. hirta* on Hep‐2 cells from human epithelioma of the larynx showed a dose dependent antitumor activity *in vitro*[45]*.* The extracts in the current investigation have also revealed antiproliferative effects on the cells that have not achieved complete attachment to the plates. This finding shows that extracts from the plant have the potential to serve as anticancer agents. The promotion of the vitality of the monkey kidney cells particularly by the crude extract of *E. hirta* implies that the administration of plant extracts in the crude form might be safer than the fractionated or purified forms. This work justifies the use of these plants particularly in their crude form for treating diseases by herbal practitioners.

## Conclusions

It might be concluded from the foregoing that extracts of both *E. hirta* and *R. vomitoria* possess antifilarial properties. Of the *E. hirta* extracts, that of ethyl acetate was found to be the most effective whilst the crude extract was the least toxic to monkey kidney cell lines. It is recommended that further efficacy determination of the extracts against both the mff and adult worms of the parasite and further toxicity studies be carried out.

## Abbreviations

Mel B: Melarsoprol; DMSPN: Dimethanesulphonate; OCRC: Onchocerciasis Chemotherapy Research Centre; DMSO: Dimethylsulfoxide (Merck, Darmstadt), dried grade; MEM: Eagle’s Minimum Essential Culture medium; E. hirta: *Euphorbia hirta*; R. vomitoria: *Rauvolfia vomitoria*; OD: Optical density; MTT: 3-(4, 5 dimethylthiazol-2-yl)-2 5-diphenyltetrazolium bromide; O. volvulus: *Onchocerca volvulus*; Mff: Microfilariae.

## Competing interests

The authors declare that they have no competing interests.

## Authors’ contributions

SKA: is the principal investigator, conceived and designed the work, carried out the extraction, data collection, analysis and interpretation.as part of a requirement for his PhD; PFA: supervised SKA for this work. AKN: also supervised SKA and contributed in the analysis and interpretation of data. AAS: assisted in the design of the work, arranged for the extraction, and freeze-drying of aqueous extracts. IVO: supplied the reagents, and supervised the extraction and freeze-drying of aqueous extracts in his laboratory. All authors contributed in the drafting and revision of the manuscript and gave their approval for the final version of the manuscript to be published.

## Pre-publication history

The pre-publication history for this paper can be accessed here:

http://www.biomedcentral.com/1472-6882/13/66/prepub

## References

[B1] Nakajima A The prevention of blindness - past present and future Yen Ko Hsueh Pao 1992 8 2 51 55 1299598

[B2] Narita AS Taylor HR Blindness in the tropics Med J Australia 1993 159 6 416 420 8377695 10.5694/j.1326-5377.1993.tb137921.x

[B3] Thylefors B Negrel AD Pararajasegaram R Dadzie KY Global data on blindness Bull Wld Hlth Org 1995 73 1 115 121 PMC2486591 7704921

[B4] Whitcher JP Srinivasan M Upadhyay MP Corneal blindness: a global perspective Bull Wld Hlth Org 2001 79 3 214 221 PMC2566379 11285665

[B5] Prost A The burden of blindness in adult males in the savanna villages of West Africa exposed to onchocerciasis TransRoy Soc Trop Med Hyg 1986 80 4 525 527 10.1016/0035-9203(86)90129-X 3810784

[B6] Prost A Vaugelade J Excess mortality among blind persons in the West African Savannah zone Bull Wld Hlth Org 1981 59 773 776 PMC2396104 6976238

[B7] WHO Third Report of the Expert Committee on Onhocerciasis Technical Report Series No. 752 1987 Geneva, Switzerland: World Health Organisation

[B8] WHO New light shed on the importance and care of onchocercal skin disease, TDR news No. 55 1998 12348563

[B9] Amazigo UO Detrimental effects of onchocerciasis on marriage age and breast feeding Trop Geogr Med 1994 46 5 322 325 7855922

[B10] Ovuga EB Ogwal-Okeny JW Okello DO Social anthropological aspects of onchocercal skin disease in Nebbi District, Uganda East Afr Med J 1995 72 10 649 653 8904045

[B11] Brieger WR Oshiname FO Ososanya OO Stigma associated with onchocercal skin disease among those affected near the Ofiki and Oyan Rivers in Western Nigeria Soc Sc Med 1998 47 7 841 852 10.1016/S0277-9536(98)00007-0 9722105

[B12] Pion SD Kamgno J Demanga N Boussinesq M Excess mortality associated with blindness in the onchocerciasis focus of the Mbam valley, Cameroon Ann Trop Med Parasitol 2002 96 2 181 189 10.1179/000349802125000718 12080979

[B13] Aziz MA Ivermectin versus onchocerciasis Parasitol Today 1986 2 9 233 235 10.1016/0169-4758(86)90001-3

[B14] Aziz MA Diallo S Diop IM Lariviere M Porta M Efficacy and tolerance of ivermectin in human onchocerciasis Lancet 1982 2 171 173 6123884 10.1016/s0140-6736(82)91026-1

[B15] Aziz MA Diallo S Lariviere M Diop IM Porta M Gaxotte P Ivermectin in onchocerciasis Lancet 1982 2 1456 1457 6129524 10.1016/s0140-6736(82)91350-2

[B16] Awadzi K Dadzie KY Schulz-Key H Haddock DRW Gilles HM Aziz MA The chemotherapy of onchocerciasis X: An assessment of four single dose treatment regimes of MK-933 (ivermectin) in human onchocerciasis Ann Trop Med Parasitol 1985 79 63 78 3838638

[B17] Awadzi K Dadzie KY Schulz-Key H Gilles HM Fulford AJ Aziz MA The chemotherapy of onchocerciasis XI: A double-blind comparative study of ivermectin, diethylcarbamazine and placebo in human onchocerciasis in northern Ghana Ann Trop Med Parasitol 1986 80 4 433 442 3539046 10.1080/00034983.1986.11812044

[B18] Awadzi K Attah SK Addy ET Opoku NO Quartey BT The effects of high-dose ivermectin regimens on *Onchocerca volvulus* in onchocerciasis patients Trans Roy Soc Trop Med Hyg 1999 93 189 194 10.1016/S0035-9203(99)90305-X 10450448

[B19] Awadzi K Boakye DA Edwards G Opoku NO Attah SK Osei-Atweneboana MY Lazdins-Helds JK Ardrey EA Addy ET Quartey BT Ahmed K Boatin BA Soumbey-Alley EW An investigation of persistent microfilaridermias despite multiple treatments with ivermectin, in two onchocerciasis-endemic foci in Ghana Ann Trop Med Parasitol2004a 98 3 231 249 10.1179/000349804225003253 15119969

[B20] Awadzi K Attah SK Addy ET Opoku NO Quartey BT Lazdins-Helds JK Ahmed K Boatin BA Boakye DA Edwards G Thirty-month follow-up of sub-optimal responders to multiple treatments with ivermectin, in two onchocerciasis-endemic foci in Ghana Ann Trop Med Parasitol 2004 98 4 359 370 10.1179/000349804225003442 15228717

[B21] Awadzi K Edwards G Opoku NO Ardrey AE Favager S Addy ET Attah SK Yamuah LK Quartey BT The safety, tolerability and pharmacokinetics of levamisole alone, levamisole plus ivermectin, and levamisole plus albendazole, and their efficacy against *Onchocerca volvulus* Ann Trop Med Parasit 2004 98 6 595 614 10.1179/000349804225021370 15324466

[B22] Eng JKL Prichard RK A comparison of genetic polymorphism in populations of *Onchocerca volvulus* from untreated- and ivermectin-treated patients Mol Biochem Parasitol 2005 142 193 202 10.1016/j.molbiopara.2005.01.021 15885823

[B23] Osei-Atwenebonna MY Eng JKL Boakye DA Gyapong JO Prichard RK Prevalence and intensity of *Onchocerca volvulus* infection and efficacy of ivermectin in endemic communities in Ghana: a two-phase epidemiological study Lancet 2007 2369 2021 2029 10.1016/S0140-6736(07)60942-8 17574093

[B24] Osei-Atweneboana MY Awadzi K Attah SK Boakye DA Gyapong JO Prichard RK Phenotypic evidence of emerging ivermectin resistance in *Onchocerca volvulus* PLoS Negl Trop Dis 2011 5 e998 10.1371/journal.pntd.0000998 21468315 PMC3066159

[B25] Adedapo AA Shabi OO Adedokun OA Anthelmintic efficacy of the aqueous crude extract of *Euphorbia hirta* Linn in Nigerian dogs Vet Arhiv 2005 75 1 39 47

[B26] Adjanohoun E Ahyi MRA Ake Assi L Dramane K Elewude JA Fadoju SO Gbile ZO Goudote E Johnson CLA Keita A Morakinyo O Ojewole JAO Olatunji O Sofowora EA Traditional Medicine and Pharmacopoeia: Contribution to Ethnobotanical and Floristic studies in western Nigeria 1991 Lagos: Organization of African Unity’s Scientific, Technical and Research Commission 308 354

[B27] Ali M Textbook of Pharmacognosy 1998 2 New Delhi: CBS. Publishers and Distributors 518

[B28] TTC/CSIR Ghana Herbal Pharmacopoeia 1992 1 Accra, Ghana: Policy Research and Strategic Planning Institute (PORSPI) of the Council for Scientific and Industrial Research 205

[B29] Sofowora A Medicinal Plants and Traditional Medicine in Africa 1993 2 Ibadan, Owerri, Kaduna and Lagos: Spectrum Books Ltd 289

[B30] Mshana NR Abbiw DK Addae-Mensah I Adjanouhoun E Ahyi MRA Ekpere JA Enow-Orock EG Gbile ZO Noamesi GK Odei MA Odunlami H Oteng-Yeboah AA Sarpong K Sofowora A Tackie AN Traditional Medicine and Pharmacopoeia: Contribution to the revision of ethnobotanical and floristic studies in Ghana 2000 1 Accra: OAU/STRC document published by the Institute for Scientific and Technical Information 920

[B31] Moundipa PF Melanie Flore KG Bilong Bilong CF Bruchhaus I *In vitro* amoebicidal activity of some medicinal plants of the Bamun Region (Cameroon) Afr J Trad Compl Altern Med 2005 2 2 113 121

[B32] Ogueke CC Ogbulie JN Okoli CI Anyanwu N Antibacterial activity and toxicological potential of crude ethanolic extracts of *Euphorbia hirta* J Amer Sc 2007 3 3 11 16

[B33] Kilian HD Jahn K Kraus L Büttner DW *In vivo* and *in vitro* effects of extracts from *Cassia aubrevillei* in onchocerciasis Acta Leiden 1990 59 1&2 365 371 2378217

[B34] Titanji VPK Evehe MS Ayafor JF Kinbu SF Novel *Onchocerca volvulus* filaricides from *Caraca procera*, *Polyalthia suaveolens and Pachypodanthium staudtii* Acta Leiden 1990 59 1&2 377 382 2378219

[B35] Ghosh M Babu SP Sukul NC Mahato SB Antifilarial effect of two triterpenoid saponins isolated from *Acacia auriculiformis* Indian J Exp Biol 1993 31 7 604 606 8225417

[B36] Singh R Khan NU Singhall KC In vitro antifilarial activity of Sencio nudicaulis Buch. Ham. Effect on Setaria cervi (Nematoda Filarioidea) Indian J Physiol Pharmacol 1996 40 231 236 8950138

[B37] Singh R Singhal KC Khan NU Exploration of antifilarial potential and possible mechanism of action of the root extracts of *Saxifraga stracheyion* on cattle filarial parasite *Setaria cervi* Phytother Res 2000 14 1 63 66 10.1002/(SICI)1099-1573(200002)14:1<63::AID-PTR567>3.0.CO;2-5 10641054

[B38] Singhal KC Sharma S Mehta BK Antifilarial activity of *Centratherum anthelminticum* seed extracts on *Setaria cervi* Indian J Exp Biol 1992 30 546 548 1506041

[B39] Mishra V Khan NU Singhal KC Potential antifilarial activity of fruit extracts of *Ficus recemosa* (Linn.) against *Setaria cervi in vitro* Indian Biol2005a 43 4 346 350 15875719

[B40] Mishra V Parveen N Singhal KC Khan NU Antifilarial activity of *Azadirachta indica* on cattle filarial parasite *Setaria cervi* Fitoterapia 2005 76 1 54 61 10.1016/j.fitote.2004.10.010 15664463

[B41] Comley JCW Titanji VPK Ayafor JF Singh VK *In vitro* antifilarial activity of some medicinal plants Acta Leiden 1990 59 1&2 361 363 2378216

[B42] Hashemi SR Zulkifli I Hair Bejo M Farida A Somchit MN Acute toxicity study and phytochemical screening of selected herbal aqueous extract in broiler chickens Int J Pharmacol 2008 4 5 352 360 10.3923/ijp.2008.352.360

[B43] Otsyina HR Toxicological evaluation of Cryptolepis sanguinolenta, Momordica charantia and Euphorbia hirta in rats. PhD thesis 2010 Kwame Nkrumah University of Science and Technology, College of Health Sciences Series 4931

[B44] Brindha D Saroja S Jeyanthi GP 2010 Protective potential of *Euphorbia hirta* against cytotoxicity induced in hepatocytes and a HepG2 cell line J Basic Clin Physiol Pharmacol 2010 21 4 401 413 21305854 10.1515/jbcpp.2010.21.4.401

[B45] Raja Sidambaram R Dinesh MG Jayalakshmi ET An *in vitro* study of cytotoxic activity of *Euphorbia hirta* on Hep2 cells of human epithelioma of larynx Intern J Pharmacy Pharmaceutical Sc 2011 3 Suppl 3 ISSN- 0975–1491

